# Improved Dewaterability of Waste Activated Sludge by Fe(II)-Activated Potassium Periodate Oxidation

**DOI:** 10.3390/ijerph192214726

**Published:** 2022-11-09

**Authors:** Hong Xiao, Qing Liu, Yingjun Wang, Ying Zhu, Dexin Fang, Ganxue Wu, Zhenxing Zeng, Hong Peng

**Affiliations:** College of Environmental Sciences, Sichuan Agricultural University, Chengdu 611130, China

**Keywords:** sludge dewaterability, ferrous ion, potassium periodate, extracellular polymeric substances

## Abstract

Fe(II)-activated potassium periodate (KIO_4_) oxidation was used to improve the dewaterability of waste-activated sludge for the first time. Compared with those of raw sludge, the capillary suction time (CST), specific resistance filtration (SRF), and water content of filter cake (W_C_) of sludge treated using the Fe(II)/KIO_4_ process under the optimal conditions (i.e., the initial pH = 6.8, KIO_4_ dose = 1.4 mmol/g volatile suspended solids, Fe(II)/KIO_4_ molar ratio = 1.2) decreased by 64.34%, 84.13%, and 6.69%, respectively. For conditioned sludge flocs, the Zeta potential and particle size were increased, and hydrophilic proteins in extracellular polymeric substances (EPS) were partly degraded, accompanied by the transformation of tightly bound EPS into soluble EPS and the conversion of dense sludge flocs into loose and porous ones. During Fe(II)/KIO_4_ oxidation, Fe(IV) and the accompanying ^•^OH were determined as the predominant reactive species and the underlying mechanism of sludge EPS degradation was proposed. This work provides a prospective method for conditioning the sludge dewaterability.

## 1. Introduction

Wastewater treatment plants produce massive waste activated sludge (WAS) with moisture content higher than 99%. Sludge dewatering is an imperative procedure to reduce the sludge volume and subsequently to save on the cost of sludge transportation and disposal. The efficiency of sludge dewatering is largely dependent on the extracellular polymeric substances (EPS) [1,2,3,4,5,6]. Destroying the EPS was deemed to be capable of improving the sludge dewatering performance. Herein, advanced oxidation processes (AOPs) have received much attention due to their efficiency in degrading EPS [7].

Traditional AOPs, such as Fenton [8] and Fenton-like [9] AOPs, have proven to be able to destroy the EPS due to the generation of hydroxyl radicals (^•^OH). Sulfate radical (SO_4_^•−^)-based AOPs seem to perform better due to SO_4_^•−^ having a higher redox potential (2.5–3.1 V) than ^•^OH (1.8–2.7 V) [10,11]. More recently, periodate-based AOPs have attracted increasing attentions for the degradation of aqueous contaminants [12,13,14,15,16,17,18,19,20,21,22,23,24]. Notably, Zong et al. [17] reported that KIO_4_ is readily activated by Fe(II) under acidic conditions, resulting in the enhanced abatement of organic contaminants, with the decay ratios of the selected pollutants even exceeding those in the Fe(II)/peroxymonosulfate (PMS) and Fe(II)/peroxydisulfate (PDS) processes under identical conditions. Thus, we are inspired to use Fe(II)-activated KIO_4_ oxidation to degrade EPS in this work, aiming to improve the dewaterability of WAS. To our best knowledge, Fe(II)-activated KIO_4_ oxidation with regards to EPS degradation has not yet been reported.

As a new strategy to improve sludge dewaterability, there are two reasons for requiring an in-depth and systematic study. One point is the optimal operational conditions of the Fe(II)/KIO_4_ oxidation system. Operational conditions influence the oxidation extent of the EPS, which is directly related to sludge dewatering performance. It is known that increasing oxidation capability promotes the degradation of the EPS. However, it is a misconception that the more the EPS degrades, the better the sludge dehydration performs. For instance, Yu et al. [6] has corroborated that hydroxylamine enhanced the Fenton (Fenton-HA) system which elevated the oxidation capability, whereas it also deteriorated sludge dewaterability. Thus, determining the optimal operational condition is of great significance. The other reason is the underlying mechanism responsible for EPS degradation in a Fe(II)/KIO_4_ oxidation matrix. From the literatures, reactive species, such as iodyl (^•^IO_3_), ^•^OH, superoxide anion (^•^O_2_^−^), singlet oxygen (^1^O_2_), and Fe(IV), may be generated during KIO_4_ oxidation. Lee et al. [25] attributed KIO_4_ oxidation on sludge EPS to ^•^IO_3_ and ^•^OH. Differently, ^•^IO_3_ was excluded as a reactive species in the Fe(II)/KIO_4_ process by Zong et al. [17]. Bokare and Choi [26] declared the generation of ^1^O_2_ in an alkaline KIO_4_ solution and monitored the reactivity of ^1^O_2_ in furfuryl alcohol degradation. Wang et al. [16] reported that ^1^O_2_ is the dominant free radical in the treatment of bisphenol AF by the FeS activated KIO_4_ system. However, the role of ^1^O_2_ in organic degradation is still quite controversial [27,28]. Noteworthily, Zong et al. [17] demonstrated that high-spin Fe(IV) and its accompanying ^•^OH accounted for the rapid removal of emerging contaminants in Fe(II)/KIO_4_ system. Altogether, it can be concluded that the reactive species and their roles vary under different circumstances. An in-depth understanding of the reactive species involved can provide implications for optimizing the reaction conditions. Furthermore, the underlying mechanism of Fe(II)-activated KIO_4_ oxidation for sludge dewaterability improvement needs to be clarified.

It is worth noting that although both this study and the research by Zong et al. [17] adopted Fe(II)-activated KIO_4_ oxidation to degrade organic matters, their application circumstances are different, i.e., one is for abatement of organic contaminants in acidic solutions, and the other is for degradation of EPS in sludge matrices. Reaction performance and mechanisms under different circumstances may be different. Moreover, achieving the highest degradation efficiency of organic matters may not mean an ideal outcome in this study. Therefore, the objectives of this study focused on (1) determining the optimal conditions (i.e., the initial pH, molar ratio of Fe (II) to KIO_4_, and KIO_4_ concentration) for sludge dewatering enhancement in an Fe(II)/KIO_4_ system, and (2) elucidating the underlying mechanism by which WAS dewaterability is improved.

## 2. Materials and Methods

### 2.1. Materials

The raw sludge was obtained from a thickening tank of a sewage treatment plant located at Wenjiang district, Chengdu city, China. The raw sludge samples were stored at 4 °C before usage, and all batch experiments were completed within one week. The main properties of raw sludge are listed in Table 1.

Ferrous sulfate heptahydrate (FeSO_4_·7H_2_O), sodium hydroxide (NaOH), potassium sodium tartrate tetrahydrate (C_4_H_4_KNaO_6_·4H_2_O), anhydrous sodium carbonate (Na_2_CO_3_), copper sulfate pentahydrate (CuSO_4_·5H_2_O), potassium bromide (KBr), sulfuric acid (H_2_SO_4_), glucose (C_6_H_12_O_6_), tert-butyl alcohol (TBA), phenol, and sodium acetate (CH_3_COONa) were purchased from Chengdu Kelong Chemical Reagent Co., Ltd. (Sichuan, China). Potassium periodate (KIO_4_), bovine albumin (BSA), Folin–phenol reagent, ascorbic acid (AA), L-histidine, nitro blue tetrazolium chloride (NBT), phenol, and methanol (MeOH) were obtained from Shanghai Aladdin Biological Technology Co., Ltd. (Shanghai, China). All chemicals were analytically pure and used without further purification.

### 2.2. Experimental Design

Fe(II)/KIO_4_ oxidation was designed to improve the dewaterability of WAS. A series of batch tests were carried out in 300 mL beakers. The beaker was continuously stirred in a thermostatic magnetic stirrer at 25 °C and 150 rpm. CST, SRF, and W_C_ were selected as representative indicators for sludge dewatering performance. To explore the effect of the initial pH on the sludge dewatering performance, the initial pH was adjusted to 3.0, 4.0, 5.0, 6.8, 8.0, and 9.0 while keeping the KIO_4_ dose (at 1.4 mmol/g VSS) and Fe(II)/KIO_4_ molar ratio (1.2) unchanged. Similarly, with the Fe(II)/KIO_4_ molar ratio fixed at 1.2 and the initial pH maintained at 6.8, the effect of KIO_4_ dose (0.6, 0.8 1.0, 1.2, 1.4, 1.6, and 1.8 mmol/g VSS) on the sludge dewatering performance was investigated. To determine the effect of Fe(II)/KIO_4_ molar ratio on the sludge dewaterability, the Fe(II)/KIO_4_ molar ratio was set as 0.0, 0.3, 0.6, 0.9, 1.2, 1.5, and 1.8 under the conditions of a KIO_4_ dose of 1.4 mmol/g VSS and an initial pH of 6.8. For all experiments, the reaction time lasted for 20 min. All experiments were repeated three times.

### 2.3. Analyses

#### 2.3.1. Determination of Sludge Dewaterability

SRF and CST are both evaluation indexes of sludge filtration and dewatering performance, while W_C_ is an index reflecting the effectiveness of sludge dewatering. The smaller the values of SRF, CST and W_C_, the better the dewatering and filtration performance of the sludge. The CST of sludge was measured by CST instrument (DP-MT) equipment. The SRF was measured by the vacuum filtration method: the Buchner funnel was filled with the sludge suspension of 100 mL, and a constant pressure of 0.06 MPa was applied using a vacuum pump. First, start the stopwatch then, when the filtration starts, the corresponding filtration volume in the measuring cylinder is recorded every 10 s. As the filtration speed slows down, the interval time of the record is increased, and the filter volume is recorded every 1 min. The filtration was stopped when there was no filter drop. The vacuum valve needs to be continuously adjusted throughout the experiment to ensure that the experimental pressure is constant. The *SRF* (m/kg) of the sludge was calculated by Equation (1) [29]:

*SRF* = 2*PA*^2^*b*/(μω) (1)

where *P* (kg/m^2^) is the pressure applied; *A* (m^2^) is the filter area; μ (kg·s/m^2^) is the KV, ω (kg/m^3^) denotes dry solid weight per unit volume sludge on the filtrate media; and *b* is the time-to-filtration ratio, which is the slope of the curve that is obtained by plotting the ratio of the time of filtration to the volume of filtrate (*t*/*V*) versus the filtrate volume (*V*).

W_C_ obtained during SRF test was determined by standard methods [30].

#### 2.3.2. Sludge Properties

The pH value was measured with a pH meter (pHs-3C). Particle size and particle size distribution were determined by a laser particle size analyzer (Malvern Master sizer 2000). The Zeta potential of the sludge supernatant was measured using a Malvern potential analyzer (Malvern Zeta sizer Nano ZS90). In addition, the physical surface morphology changes of sludge flocs samples were observed by a scanning electron microscope (SEM) (ZEISS Sigma 300). The surface functional groups of sludge flocs were determined by Fourier transform infrared spectroscopy (FT-IR) (Nicolet IS5, Thermo Fisher Scientific, Waltham, MA, USA).

EPS extraction was conducted according to a thermal extraction method [31]. A concise description is as follows: A 15 mL sludge sample was centrifuged at 4000× *g* at 4 °C for 15 min. The supernatant was collected and denoted SB-EPS. The pellet was resuspended in 15 mL of 0.05% NaCl using a vortex mixer, heated at 70 °C for 1 min, and subsequently centrifuged at 4000× *g* at 4 °C for 10 min. The collected supernatant was considered LB-EPS. The residual pellet was resuspended in a 0.05% NaCl solution to its original volume, incubated at 60 °C in a water bath for 30 min, and centrifuged at 4000× *g* at 4 °C for 15 min to collect TB-EPS. The protein and polysaccharide concentration in EPS were determined using the Lowry method or the phenol-sulfuric acid method [32].

## 3. Results and Discussion

### 3.1. Optimization of Sludge Dewatering Conditions

#### 3.1.1. Effects of Initial pH on Sludge Dewaterability

**Figure 1:**
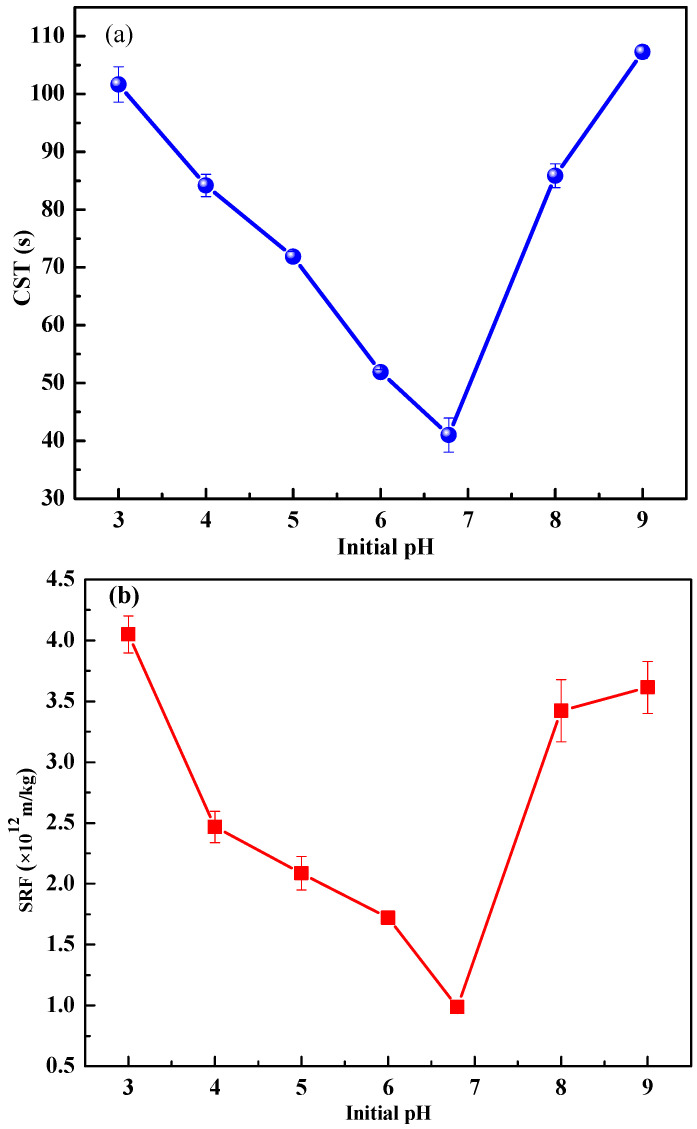

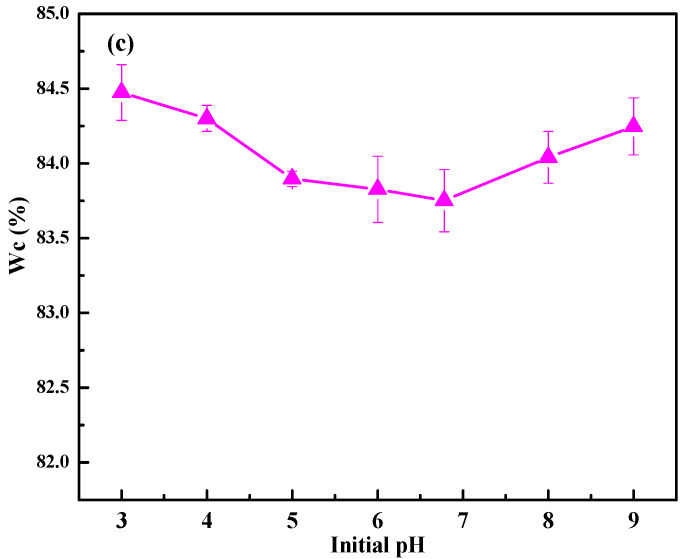
Effect of initial pH on performance indices ((**a**) CST, (**b**) SRF, and (**c**) W_C_) of sludge dewatering. Conditions: KIO_4_ dose = 1.4 mmol/g VSS; Fe(II)/KIO_4_ molar ratio = 1.2.

The initial pH value always plays a key role in AOPs, which will affect the sludge dewaterability adjustment by Fe(II) activated KIO_4_ oxidation. Figure 1 depicts the effects of the initial pH on the performance indices of sludge dewatering.

As shown in Figure 1a, the CST of the treated sludge received its minimum value (47.55 s) at the initial pH of 6.8, which was the pH of the raw sludge. Similarly, the SRF and W_C_ of treated sludge with no pH adjustment gained minimum values of 0.99 × 10^12^ m/kg (Figure 1b), and 83.75% (Figure 1c), respectively. Interestingly, as the initial pH decreased from 6.8 to 3.0, the CST, SRF, and W_C_ increased to 101.65 s, 4.05 × 10^12^ m/kg, and 84.47%, respectively, which signified deterioration in the sludge dewaterability. As it is known that a lower pH in AOPs always results in a higher production of active species, and, consequently, a higher degradation of organic contaminants. During recent decades, EPS has been reckoned as a key factor in hampering sludge dewatering. Many studies endeavored to degrade EPS as much as possible so as to improve sludge dewaterability. However, in this study, more degradation of EPS at an initial pH lower than 6.8 exhibited negative effects on sludge dewaterability. A plausible explanation is the layered structure of EPS and its hydrophilic/hydrophobic characteristics. As Yu et al. [6] reported, EPS were degraded layer by layer in sludge oxidation, and different EPS layers showed quite different hydrophilic/hydrophobic organic distributions. The surface EPS layer possesses a relatively higher hydrophobicity, whereas the inner EPS layer has a relatively higher hydrophilicity. Excessive destruction of the surface hydrophobic EPS and exposure of the inner hydrophilic EPS elevated the energy barrier among sludge flocs, which increased the bound water content of the sludge and resultantly deteriorated the sludge dewaterability [6,33,34]. While increasing the initial pH from 6.8 to 9.0, the sludge dewatering performance also showed a downward trend. The CST, SRF, and W_C_ increased to 107.25 s, 3.61 × 10^12^ m/kg, and 84.25%, respectively. This can be explained as follows: In alkaline conditions, Fe(II) easily reacts with OH^−^ and reduces the amount of effective activator in the reaction system. Moreover, Fe^2+^ and Fe^3+^ react easily with OH^−^ to form Fe(OH)_2_ and Fe(OH)_3_ colloids, which increase the viscosity of sludge and affect its filtration ability. As a result, sludge dewatering becomes more difficult [32]. To sum up, an initial pH that is too low or too high is not conducive to improving the sludge dewaterability, and the initial pH of 6.8 is optimal. This result implied that there is no need to adjust the initial pH during sludge conditioning by Fe(II)/KIO_4_ oxidation.

#### 3.1.2. Effects of KIO_4_ Dose on Sludge Dewaterability

**Figure 2:**
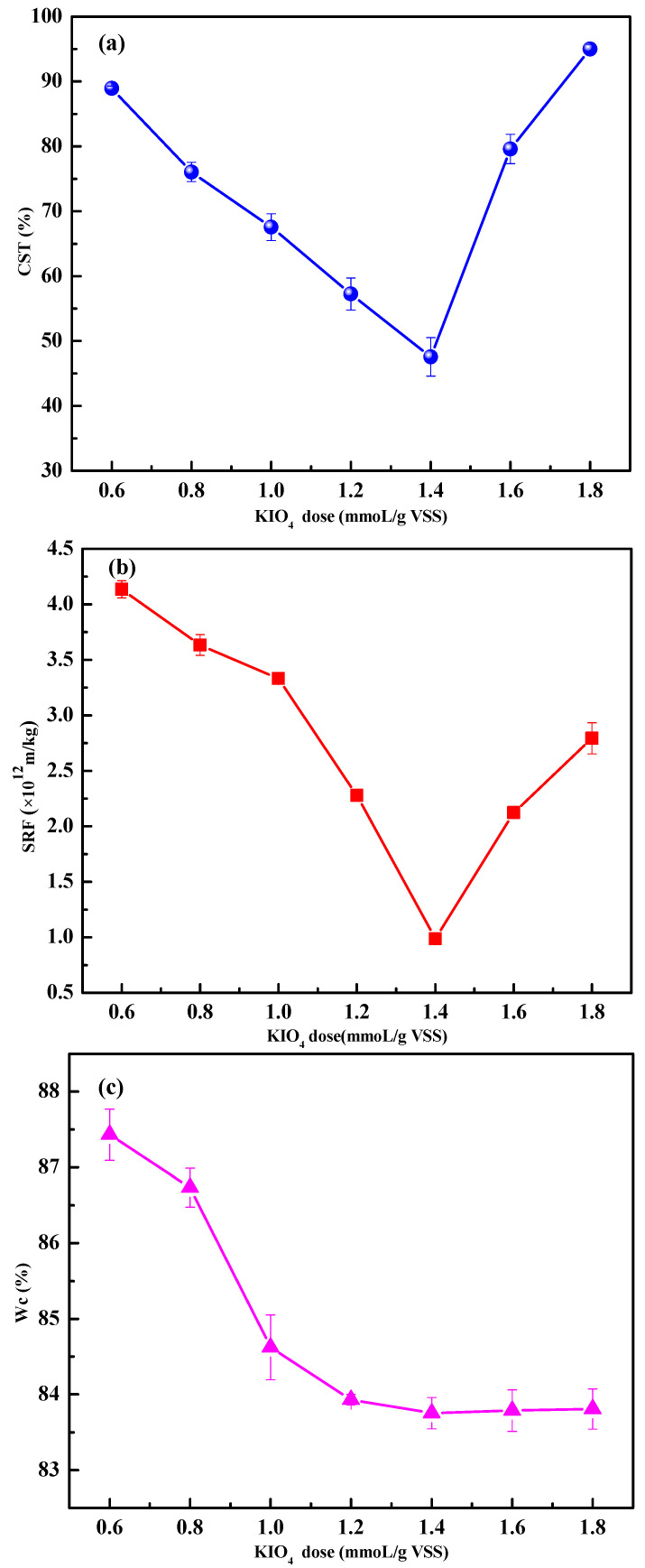
Effect of KIO_4_ dose on performance indices of sludge dewatering. (**a**) CST, (**b**) SRF, and (**c**) W_C_. Conditions: Initial pH = 6.8; Fe(II)/KIO_4_ molar ratio = 1.2.

Figure 2 shows the effects of KIO_4_ dose pH on performance indices of sludge dewatering.

With the KIO_4_ dose increased from 0.6 to 1.4 mmol/g VSS, the CST, SRF, and W_C_ obviously decreased from 88.95 s, 4.14 × 10^12^ m/kg and 87.43%, to 47.55 s, 0.99 × 10^12^ m/kg and 83.75%, respectively. This implied that more reactive species were generated with the increase in KIO_4_ dose, which led to an enhanced oxidation of sludge flocs [13]. As the KIO_4_ dose further increased to 1.8 mmol/g VSS, CST and SRF increased instead; meanwhile, W_C_ maintained stable. On the one hand, an excess of IO_4_^−^ will react with reactive free radicals (Equation (2)) [35]. On the other hand, due to the decrease in flocculation activity of overoxidized sludge, the sludge filterability is reduced and the dehydration is deteriorated [36].

IO_4_^−^ + ^•^OH → OH^−^ + ^•^IO_4_ (2)

#### 3.1.3. Effects of Fe(II)/KIO_4_ Molar Ratio on Sludge Dewaterability

**Figure 3:**
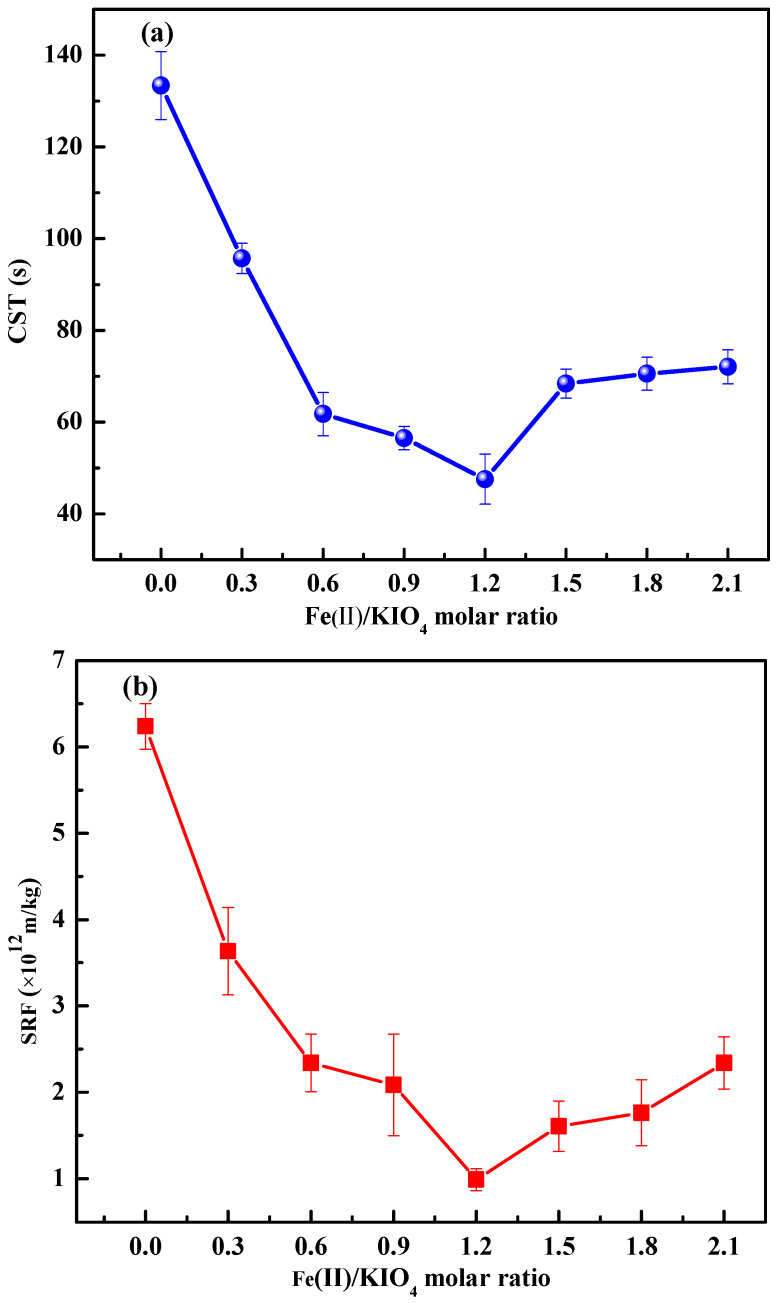

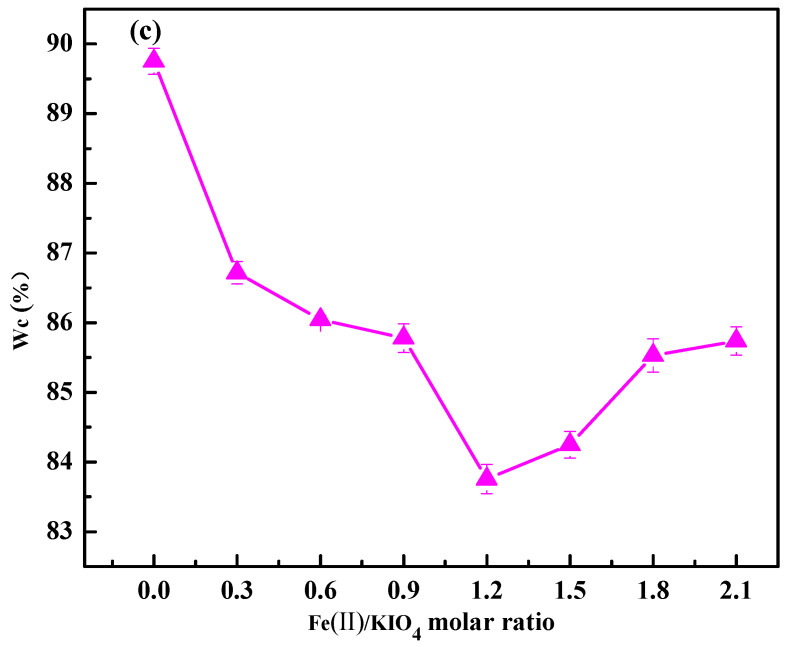
Effect of Fe(II)/KIO_4_ molar ratio on performance indices of sludge dewatering. (**a**) CST, (**b**) SRF, and (**c**) W_C_. Conditions: Initial pH = 6.8; KIO_4_ dose = 1.4 mmol/g VSS.

Figure 3 describes the effects of Fe(II)/KIO_4_ molar ratio on performance indices of sludge dewatering.

As the Fe(II)/KIO_4_ molar ratio increased from 0 to 1.2, the CST, SRF, and W_C_ obviously decreased from 133.35 s, 6.24 × 10^12^ m/kg and 89.75%, to 47.55 s, 0.99 × 10^12^ m/kg and 83.75%, respectively. The reason for the improvement of sludge dewatering performance is that Fe^2+^, as an initiator, activates IO_4_^−^ to generate free radicals, which accelerates the disintegration of sludge flocs. When the Fe(II)/KIO_4_ molar ratio was lower than 1.2, insufficient ferrous ions were not capable of forming enough reactive species and, therefore, the oxidation of sludge flocs was greatly limited [37]. Whereas Fe(II)/KIO_4_ molar ratio exceeded 1.2, the dewatering performance of the sludge became poor, which was mainly due to the fact that the excess ferrous consumed the reactive species produced. For instance, excessive Fe(II) would result in a competitive consumption of Fe(IV) (*k*_(Fe(IV)+Fe(II))_ = 1.4 × 10^5^ M^−1^ s^−1^) [17].

### 3.2. Effects of Fe(II)/KIO_4_ Process on Sludge Properties

#### 3.2.1. Zeta Potential and Particle Size Distribution

Zeta potential can reflect the stability of the sludge colloid matrix to a certain extent and, therefore, is listed as one of the key indicators to evaluate the dewatering performance of sludge [38].

**Figure 4:**
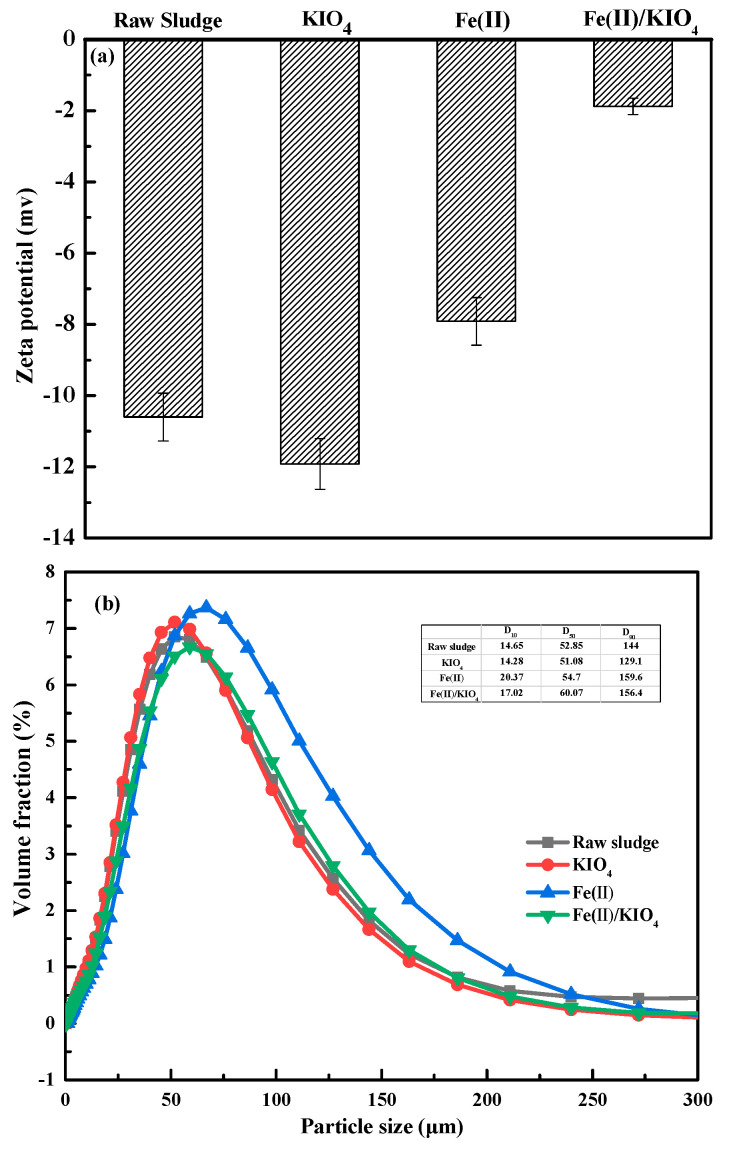
Variations of (**a**) Zeta potential and (**b**) particle size distribution of sludge flocs after treatment by different processes. Conditions: Initial pH = 6.8; KIO_4_ dose = 1.4 mmol/g VSS, Fe(II) dose = 1.68 mmol/g VSS.

As shown in Figure 4a, the Zeta potential of raw sludge was −10.60 mV, which was due to the existence of carboxylic acid and phosphate groups in the EPS [39]. As a comparison, the Zeta potential of the sludge treated by KIO_4_ alone changed to −11.92 mV, which brought about an increased electrostatic repulsion and, hence, was detrimental to the improvement of dewatering performance. After treatment with ferrous iron alone, the Zeta potential increased to −7.91 mV, which may be caused by the neutralization of negative charges incurred by Fe^2+^ and its oxidization product (Fe^3+^). Meanwhile, the Zeta potential of the sludge treated by the Fe(II)/KIO_4_ oxidation system increased to −1.87 mV, which boosted the coagulation of sludge flocs and significantly improved the dewatering performance of sludge. The increase in Zeta potential may be ascribed to the decomposition of negatively charged organic matter in sludge EPS and neutralization of negative charges by positive-charged iron ions.

The particle size is usually considered another key factor affecting sludge dewatering. As displayed in Figure 4b, the medium value of particle size (D_50_) of sludge flocs was reduced from 52.85 to 51.08 μm after treatment by KIO_4_ alone. The reduction in D_50_ can be explained by the release of some hydrophilic groups in the sludge EPS subjected to the oxidation of KIO_4_. One accompanying negative effect is the blockage of the filtration channel in sludge flocs. Once Fe^2+^ was added, the negative charges on the surface of the sludge flocs were neutralized, and the sludge flocs quickly aggregated into larger sludge particles with a D_50_ being 64.7 μm. In regards to the Fe(II)/KIO_4_ process, the oxidation effect tended to make the sludge flocs smaller. The oxidation of active species led to a decrease in hydrophilic organic compounds such as proteins. On the other hand, the flocculation effect of iron ions drove the sludge flocs to become larger. As a compromise, the D_50_ of sludge flocs treated by Fe(II)/KIO_4_ process became 60.07 μm, which was larger than the D_50_ of raw sludge (52.85 μm) and helped to improve the sludge dewaterability.

#### 3.2.2. FT-IR Spectra and SEM Analyses

To further understand the impact of Fe(II)/KIO_4_ oxidation on sludge dewaterability, the changes in functional groups on sludge flocs were analyzed by FT-IR spectra.

**Figure 5:**
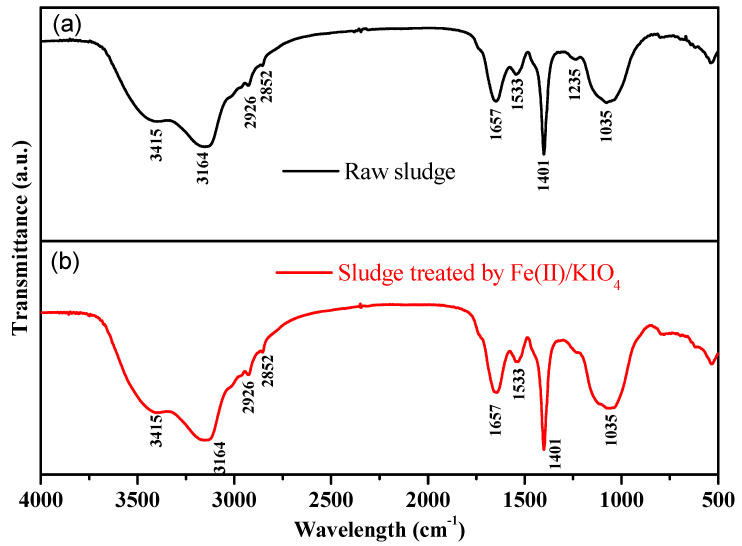
FT-IR spectra of (**a**) raw sludge and (**b**) sludge treated by Fe(II)/KIO_4_ process. Conditions: Initial pH = 6.8, KIO_4_ dose = 1.4 mmol/g VSS, Fe(II)/KIO_4_ molar ratio = 1.2.

As exhibited in Figure 5a, the peaks around 3415 cm^−1^ and 3164 cm^−1^, correspond to O-H stretching vibrations (mainly present in polysaccharides and phenols [40]). The peaks around 2926 cm^−1^ and 2852 cm^−1^, are related to the symmetric and asymmetric stretching vibrations of CH_2_ in lipids. The amide I band at 1657 cm^−1^ is mainly related to C=O and C-N stretching associated with proteins. The swelling vibration of N-H and C-N on amide II in the protein is related to the peak near 1533 cm^−1^. The peaks near 1401 cm^−1^, 1235 cm^−1^, and 1035 cm^−1^, corresponds to the bending vibration and C-O stretching vibration in the face of phenolic O-H, the asymmetric stretching vibration of C-O-C in ester, and the stretching vibration of C-O in polysaccharide, respectively [41]. As presented in Figure 5b, after treatment by Fe(II)/KIO_4_, the peak at 1235 cm^−1^ almost disappeared, suggesting the decomposition of the corresponding organic matter. Meanwhile, the relative intensities of typical peaks located around 1657 cm^−1^, 1533 cm^−1^, and 1035 cm^−1^, decreased, indicating that proteins and polysaccharides on the surface of sludge flocs were partly degraded.

**Figure 6:**
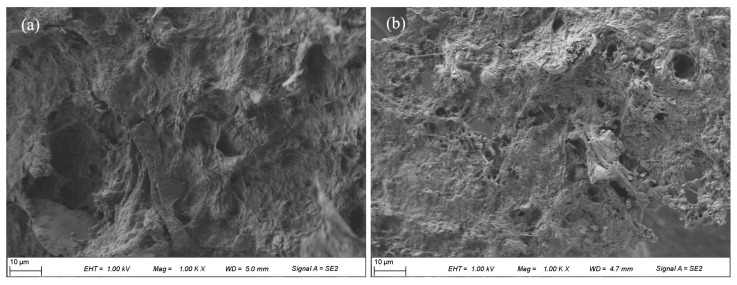
SEM of (**a**) raw sludge and (**b**) treated sludge. Conditions: Initial pH = 6.8, KIO_4_ = 1.4 mmol/g VSS, Fe(II)/KIO_4_ molar ratio = 1.2.

To intuitively analyze the influence of sludge treatment on the structure of sludge flocs, SEM analyses were conducted. As shown in Figure 6a, the raw sludge flocs were dense with few pores. This structural characteristic was embodied with low porosity and few filter channels. As a result, water is difficult to separate from the sludge [42]. Therefore, the SRF and CST of raw sludge were maintained at a high level. After treatment by the Fe(II)/KIO_4_ process, the dense structure of the sludge flocs transformed to become loose and porous (Figure 6b). The sludge drainage passages for more water permeation were thus reconstructed, which was in favor of promotion of sludge filterability [39,43].

#### 3.2.3. Polysaccharide and Protein in EPS

EPS consists of high hydrophilic substances, and those substances mainly include proteins and polysaccharides [6]. EPS is highly charged polymers that interact with water molecules to form gels, which can be divided into three parts from outside to inside, namely, the soluble EPS (S-EPS), the loosely bound EPS (LB-EPS), and the tightly bound EPS (TB-EPS) [44]. The S-EPS is weakly bound to cells or dissolved in solutions. The LB-EPS is a loose slime layer without an obvious end. The TB-EPS is bound to the cell surface tightly and stably [45].

**Figure 7:**
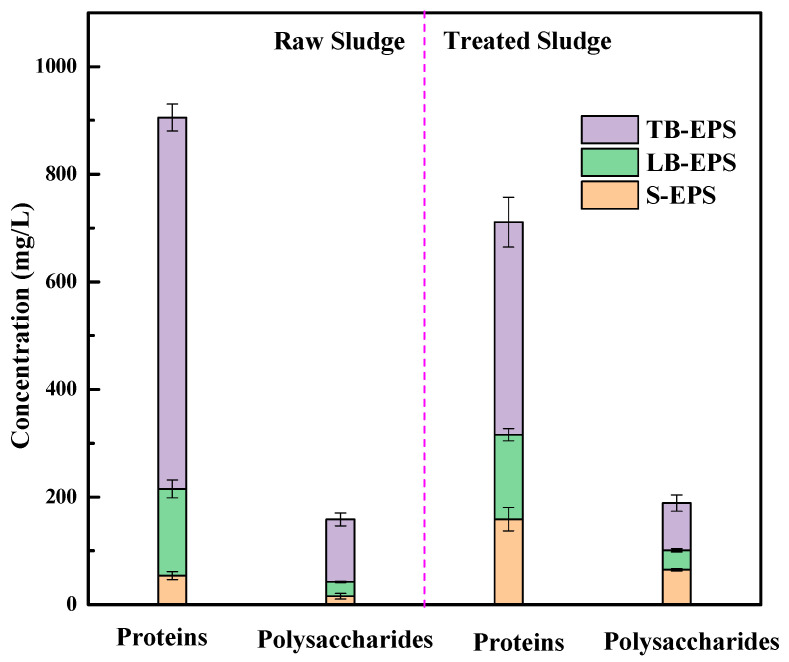
Concentrations of proteins and polysaccharides in different fractions of EPS before and after sludge conditioning. Conditions: Initial pH = 6.8, KIO_4_ dose = 1.4 mmol/g VSS, Fe(II)/KIO_4_ molar ratio = 1.2.

As shown in Figure 7, for raw sludge, the proteins concentrations in S-EPS, LB-EPS, and TB-EPS were 53.85, 161.21, and 690.56 mg/L, respectively. In regard to the treated sludge, the proteins concentration in S-EPS greatly increased to 158.85 mg/L, whereas the protein concentration in TB-EPS sharply decreased to 395.26 mg/L. This result indicated that LB-EPS was transformed into S-EPS. Similarly, it has been reported that the oxidative dissolution of EPS leads to the gradual transformation of TB-EPS to LB-EPS and subsequently to S-EPS, and the increased protein content in SB-EPS improved sludge dewatering [46,47]. As a whole, the concentration of proteins in EPS of treated sludge showed a 21.5% decline in comparison to that of raw sludge. In this study, along with the degradation of proteins, the CST and SRF decreased. This result was parallel to the finding by Wang et al. [48], who reported that proteins (including aromatic protein substances and tryptophan protein substances) are negatively correlated with sludge dewaterability. Moreover, the degradation of protein-like substances has been proven to be the key organics benefitting the improvement in sludge dewaterability [49,50].

In addition, the concentration of polysaccharides in the EPS of treated sludge exhibited a slight increase instead of decrease, which is in accordance with Liu et al. [32], who concluded that polysaccharides showed little influence on sludge dewaterability in comparison with proteins while pretreating WAS by Fe(II)-activated PMS oxidation. Similarly, Wu et al. [51] reported that high hydrophilic proteins played a more important role in sludge dewaterability than polysaccharides.

### 3.3. Reactive Species

**Figure 8:**
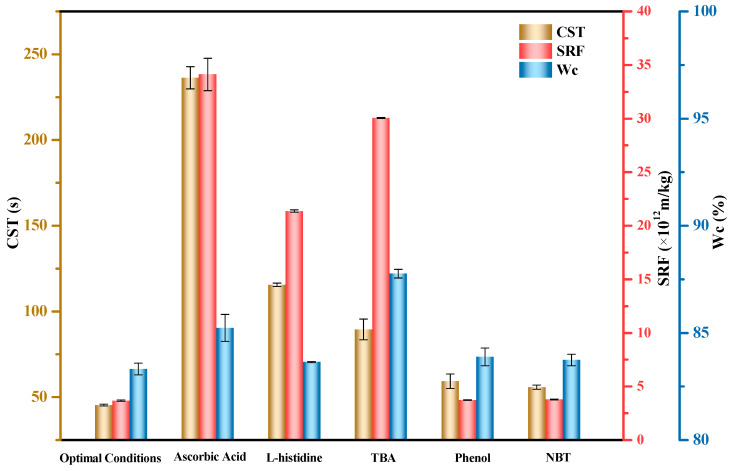
CST, SRF, and W_C_ under the cases of using different quenchers. Optimal conditions: Initial pH = 6.8, KIO_4_ dose = 1.4 mmol/g VSS, Fe(II)/KIO_4_ molar ratio = 1.2. Radical scavenger usages: 50 mM ascorbic acid, 50 mM L-histidine, 60 mM TBA, 70 mM phenol, and 12 mM NBT, respectively.

The identification of reactive species is of great importance to understand the underlying mechanism of the Fe(II)/KIO_4_ process. The possible reactive species include ^•^OH, singlet oxygen (^1^O_2_), superoxide anion radical (^•^O_2_^−^), iodate radical (^•^IO_3_), and Fe(IV) [12,17]. It has been reported that ascorbic acid (AA) has the ability of non-selective scavenging free radicals, which can be used to preliminarily test whether radical species were generated in the reaction system [52]. Moreover, TBA [53], L-histidine [54], NBT, and phenol [19] have been proven to possess strong scavenging ability on ^•^OH, ^1^O_2_, ^•^O_2_^−^, and ^•^IO_3_, respectively. Therefore, AA, TBA, L-histidine, NBT, and phenol were used in this study to identify and differentiate free radicals. Significantly, Zong et al. [17] have provided conclusive evidence for the generation of high-valent iron−oxo species (Fe(IV)) in the Fe(II)/KIO_4_ process. In more detail, Fe(H_2_O)_6_^2+^ first reacts with IO_4_(H_2_O)^−^ to generate a hydrogen bonding complex Fe(H_2_O)_6_^2+^IO_4_(H_2_O)^−^ (INT1) (Equation (3)); then, one of the six H_2_O molecules that are originally coordinated with Fe^2+^ is exchanged by IO_4_^−^ to form an inner shell complex Fe(H_2_O)_5_^2+^−OIO_3_(H_2_O)^−^ (INT2) (Equation (4)); and thereafter INT2 undergoes an oxygen atom transfer step to consequently produce Fe=O(H_2_O)_5_^2+^IO_3_(H_2_O)^−^ (i.e., Fe(IV) and IO_3_^−^) (Equation (5)). Note that Fe(IV) undergoes self-decay and subsequently generates H_2_O_2_ and Fe(III) (Equation (6)) [55]; thus, ^•^OH can be considered to be indirectly triggered by the self-decomposition of Fe(IV) and the subsequent Fenton reaction (Equation (7)). In addition, it is inferred that ^•^O_2_^−^ was generated according to Equation (8). As depicted in Figure 8, the values of three indices (i.e., CST, SRF, and W_C_) increased with the addition of AA, indicating that radical species were involved in this system. After the addition of TBA, the values of the three indices also greatly increased, suggesting that scavenging of ^•^OH produced in the Fe(II)/KIO_4_ process worsened the sludge dewaterability. It was noteworthy that the additions of NBT and phenol had a slight impact on sludge dewatering performance, indicating that ^•^O_2_^−^ and ^•^IO_3_ played a small role. In fact, the redox potential of ^•^O_2_^−^ is only −0.28 V [56,57], and its oxidative contribution to the degradation of sludge EPS was thus expected to be negligible [58]. Moreover, ^•^IO_3_ was excluded as a reactive species in the Fe(II)/KIO_4_ process [17]. The seemingly quenching effect that arose from the addition of phenol may be ascribed to the adverse impact of phenol on other reactive species. In regards to the addition of L-histidine, its effect on sludge dewaterability was slightly more obvious compared to the additions of NBT and phenol. However, ^1^O_2_ was excluded as a reactive species in the Fe(II)/KIO_4_ process based on irrefutable proofs [17]. Herein, whether L-histidine will react with Fe(IV) or KIO_4_ and resultantly poses negative influence on the oxidizing power of Fe(IV) or the generation of ^•^OH merits further investigations.

In summary, the mechanism of sludge EPS degradation by Fe(II)/KIO_4_ oxidation under optimal conditions was proposed to consist of the reactions shown in Equations (3)–(11) [17]:

Fe(H_2_O)_6_^2+^ + IO_4_(H_2_O)^−^ → Fe(H_2_O)_6_^2+^IO_4_(H_2_O)^−^ (3)

Fe(H_2_O)_6_^2+^IO_4_(H_2_O)^−^ → Fe(H_2_O)_5_^2+^-OIO_3_(H_2_O)^−^ (4)

Fe(H_2_O)_5_^2+^-OIO_3_(H_2_O)^−^ → Fe(IV) + IO_3_^−^ (5)

2Fe(IV) → 1/3H_2_O_2_ + 2Fe(III) + 1/3O_2_ (6)

Fe(II) + H_2_O_2_ → Fe(III) + ^•^OH + OH^−^ (7)

Fe(II) + IO_4_^−^ + H_2_O → Fe(III) + IO_3_^−^ + ^•^O_2_^−^ + 2H^+^ (8)

Fe(IV) + EPS → degradation products + Fe(III)(9)

^•^OH + EPS → degradation products (10)

### 3.4. Environmental Implications

Taking the effects of initial pH, KIO_4_ dose, and Fe(II)/KIO_4_ molar ratio on sludge dewaterability into comprehensive considerations, the optimal conditions are as follows: the initial pH = 6.8, the KIO_4_ dose = 1.4 mmol/g VSS, and the Fe(II)/KIO_4_ molar ratio = 1.2. Compared to those of the untreated raw sludge, the CST (47.55 s) decreased by 64.34%, SRF (0.99 × 10^12^ m/kg) decreased by 84.13%, and W_C_ (83.75%) decreased by 6.69% under the optimal conditions. It can be concluded that the dewaterability of WAS can be effectively improved by Fe(II)/KIO_4_ oxidation. As for the underlying mechanism, the role of Fe(IV) as a reaction intermediate needs to be emphasized. Although the reactivity of Fe(IV) toward organics is generally weaker than that of radical species (i.e., ^•^OH and SO_4_^•−^), the steady-state concentration of Fe(IV) (∼10^−9^ M) could be several orders of magnitude higher than those of ^•^OH and SO_4_^•−^ (∼10^−12^ M). By in situ generating the high-spin Fe(IV) species and accompanying ^•^OH, the Fe(II)/KIO_4_ process could achieve a better performance on the degradation of representative pollutants in comparison with the Fe(II)/PMS and Fe(II)/PDS processes [17]. In this work, no comparisons in terms of EPS degradation degree between Fe(II)/KIO_4_ and Fe(II)/PMS or Fe(II)/PDS were conducted; hence, we cannot claim that Fe(II)/KIO_4_ has a higher oxidizing capacity towards EPS. Additionally, excessive degradation of EPS may instead deteriorate the sludge dewatering performance [6]. It can be inferred that the Fe(II)/KIO_4_ process achieved a moderate degree of oxidation towards sludge EPS under the optimal conditions. Apart from the effectiveness in improving sludge dewatering performance, the Fe(II)/KIO_4_ process has other attractive advantages. Compared with liquid-form oxidants (e.g., H_2_O_2_, hypochlorous acid, and peracetic acid), KIO_4_, as a solid-form oxidant, is relatively stable and carries less risk during transport and storage. More importantly, there is no requirement for regulation of the initial pH, which can reduce the consumption of agents and simplify the operation. Attributed to the abovementioned advantages, Fe(II)-activated KIO_4_ oxidation has great prospect and practical value in sludge conditioning for dewatering.

## 4. Conclusions

In this study, Fe(II)-activated KIO_4_ oxidation was successfully applied to improve the sludge dewatering performance, which is closely relevant to the evolution of sludge properties. Under the optimal conditions, the CST can decrease by 64.34%, SRF can be reduced by 84.13%, and W_C_ can be lowered by 6.69%. A moderate degree of oxidation towards sludge EPS is crucial for enhancing sludge dewaterability. Herein, regulation of the initial pH of raw sludge is not essential, contributing to less consumption of agents and a simpler operation. High hydrophilic proteins played a major role, whereas polysaccharides played a negligible part in sludge conditioning. In situ generated Fe(IV) should receive special attention, not only due to its own oxidation capacity, but also due to its self-decay that can trigger the generation of ^•^OH. Fe(IV) and accompanying ^•^OH are predominant reactive species responsible for the oxidation of EPS. This work proposes a facile strategy to improve the sludge dewaterability.

## Figures and Tables

**Table 1:** Characteristics of raw sludge.

Parameter	Value
pH	6.8 ± 0.2
Water content of filter cake (Wc, %)	83.75 ± 0.21
Total suspended solids (TSS, g/L)	21.25 ± 0.3
Volatile suspended solid (VSS, g/L)	8.14 ± 0.1
Specific resistance filtration (SRF) (10^12^ m/kg)	6.24 ± 0.4
Zeta potential (mV)	−10.60 ± 0.4
Capillary suction time (CST) (s)	133.35 ± 2

## Data Availability

Not applicable.
